# Wide dispersion of B chromosomes in *Rhammatocerus brasiliensis* (Orthoptera, Acrididae)

**DOI:** 10.1590/1678-4685-GMB-2019-0077

**Published:** 2020-06-10

**Authors:** Adriana S. Melo, Geyner A.S. Cruz, Aline P. Félix, Marília F. Rocha, Vilma Loreto, Rita C. Moura

**Affiliations:** 1 Universidade de Pernambuco (UPE), Instituto de Ciências Biológicas, Laboratório de Biodiversidade e Genética de Insetos, Recife, PE, Brazil.; 2 Universidade de Pernambuco (UPE), Laboratório de Biodiversidade e Genética Evolutiva, Campus Petrolina, Petrolina, PE, Brazil.; 3 Universidade Federal de Pernambuco (UFPE), Departamento de Genética, Laboratório de Genética Animal e Humana e Citogenética, Recife, PE, Brazil.

**Keywords:** Grasshopper, supernumerary chromosome, chromosome polymorphism, ISSR, genetic connectivity

## Abstract

The grasshopper *Rhammatocerus brasiliensis* shows polymorphism of B chromosomes, but the magnitude of B-chromosome occurrence and the factors that may contribute to their dispersion in the species remain unknown thus far. The present study analyzed the occurrence and dispersion of B chromosomes in *R. brasiliensis* individuals from 21 populations widely distributed in the Brazilian Northeast. The genetic connectivity between 10 populations was verified through analysis of ISSR markers from 200 individuals. Of the 21 populations, 19 presented individuals with one B chromosome, three with two, and one with three B chromosomes. The B chromosome is of medium size and constitutive heterochromatin (CH) located in the pericentromeric region. A variant B chromosome was observed in three populations, similar in size to that of chromosome X, gap and CH, and located in the terminal region. B chromosome frequencies in different populations varied from 0% to 18,8%, mean 8,5%. The wide distribution of the B chromosome is likely a consequence of the positive gene flow among the analyzed populations. B-chromosome occurrence in populations of *R. brasiliensis* possibly follows the population genetic structure of the species and, owing to the existence of a variant, its origin may not be recent.

## Introduction

B chromosomes, also known as supernumerary chromosomes, are extra elements of the karyotype. These chromosomes do not obey the Mendelian laws of inheritance and do not present homology with the other chromosomes of the A complement (Camacho *et al.*, 2000). Moreover, they generally differ by the accumulation of repetitive DNA sequences, such as satellite DNA (Ruiz-Ruano *et al.*, 2017; Milani *et al.*, 2017a), ribosomal DNA and histone H3 (Oliveira *et al.*, 2011), U2 small nuclear RNA (Bueno *et al.*, 2013), and transposable elements (Palacios-Gimenez *et al.*, 2014). Although repetitive sequences are common in the composition of B chromosomes, genes conferring resistance to antibiotics and pathogens have also been identified (Coleman *et al.*, 2009), besides others that participate in the control of the cell cycle (Valente *et al.*, 2014; Ramos *et al.*, 2016).

The occurrence of B chromosomes is almost universal, being found in approximately 15% of the analyzed eukaryotes (Camacho, 2005). Among the animals, the insects of the order Orthoptera stand out for the great frequency of B chromosomes, being hotspots in the superfamilies Acridoidea, Grylloidea, Pyrgomorphoidea and Tetrigoidea (Palestis *et al.*, 2010). In Acridoidea, the Acrididae family is considered a hotspot representing 17,1% (Palestis *et al.*, 2010). *Locusta migratoria* (Linnaeus, 1758) and *Eyprepocnemis plorans* (Charpentier, 1825) of this family are used as models to better understand the occurrence, origin and evolution of the B chromosomes (Bueno *et al.*, 2013).

When the frequency of B chromosomes in a population is equal to or higher than 25%, it is suggested that their origin is recent (Araújo *et al.*, 2001). Contrarily, in populations where the frequency is relatively low (below 25%), the B chromosome is considered as stable or in process of elimination (Riera *et al.*, 2004). According to some authors (Camacho *et al.*, 1997, 2015; Zurita *et al.*, 1998), B chromosomes present four main life-cycle stages: 1) invasion, where a B chromosome arrives or emerges in a natural population and its frequency rises rapidly within only a few tens of generations; 2) resistance, when a B chromosome remains in the host genome through drive mechanisms; 3) near neutrality, when the frequency of B chromosomes varies randomly by genetic drift, or decreases due to negative selection in individuals with increased number of B chromosomes (this constitutes the longest stage, which can last for thousands of generations); and 4) regeneration, where the B chromosome depends on the accumulation of mutations for the emergence of novel variants that may be able to remain in the genome and substitute the former, neutralized B chromosome, reinitiating the B chromosome cycle and prolonging its evolutionary life.

For Neotropical grasshoppers, particularly those occurring in Brazil, most works performed so far have focused on the analysis of the origin and composition of B chromosomes through the mapping of different repetitive sequences, as observed for the species *Abracris flavolineata* (*2n* = 24, X0 + B and *2n* = 25, X0 + 2Bs) (Bueno *et al.*, 2013; Milani and Cabral-de-Mello, 2014; Palacios-Gimenez *et al.*, 2014; Menezes-de-Carvalho *et al.*, 2015; Milani *et al.*, 2017a,b) and *Xyleus discoideus angulatus* (*2n* = 24, X0 + B and *2n* = 25, X0 + 2Bs) (Loreto *et al.*, 2008a; Machado *et al.*, 2014; Bernardino *et al.*, 2017). For *Rhammatocerus brasiliensis*, the frequency of B chromosomes has also been analyzed in eight populations from Pernambuco and three from Bahia, states of the Brazilian Northeast, showing *2n* = 24, X0 + B and *2n* = 25, X0 + 2Bs (Loreto *et al.*, 2008a,b; Oliveira *et al.*, 2011). Regarding Neotropical species, the distribution of B chromosomes in natural grasshopper populations has not been analyzed under consideration of the genetic structure of the populations, and such studies have only been performed in Palearctic grasshoppers (Keller *et al.*, 2013; Manrique-Poyato *et al.*, 2013a,b, 2015). This kind of approach allows estimating the gene flow among individuals within and between populations, which may be a parameter directly related to the dispersion or isolation of B chromosomes (Manrique-Poyato *et al.*, 2015).

*R. brasiliensis* is widely distributed in the Northeast of Brazil, with records in the states of Rio Grande do Norte, Paraíba, Pernambuco (Assis-Pujol, 1998), and Bahia (Loreto *et al.*, 2008a). In Pernambuco, the species is found across the entire state, having been reported in 11 localities (Carbonell, 1995; Assis-Pujol, 1998; Loreto *et al.*, 2008a). Because of its wide distribution (Carbonell, 1988), *R. brasiliensis* is an interesting species for understanding the life-cycle stages of B chromosomes and how they may disperse. In this study, the following hypotheses were tested: 1) B chromosomes are dispersed among *R. brasiliensis* populations according to gene flow existing among them; 2) based on the relatively low frequency of B chromosomes in *R. brasiliensis*, as verified in different populations by Loreto *et al.* (2008b), it is expected that the B chromosome is currently in the near-neutrality stage.

With this in mind, we analyzed the occurrence, frequency and distribution of B chromosomes in *R. brasiliensis* individuals from 21 populations from the Brazilian Northeast, of which 10 were also analyzed regarding population genetic structure. In addition, the position of the constitutive heterochromatin (CH) was established in the B chromosomes of the species.

## Material and Methods

### Sampling

**Figure 1:**
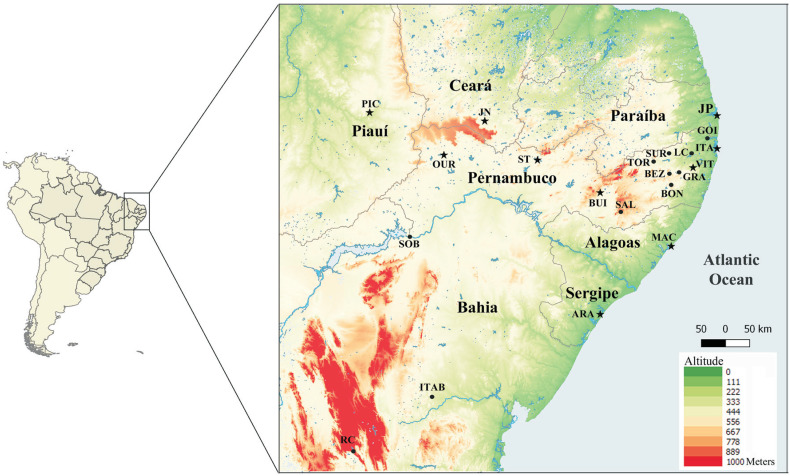
Partial map of the Brazilian Northeast indicating the sampling locations of *Rhammatocerus brasiliensis* populations. Aracaju (ARA) – SE; Maceió (MAC) – AL; João Pessoa (JP) – PB; Itamaracá (ITA) – PE; Vitória de Santo Antão (VIT) – PE; Goiana (GOI) – PE; Gravatá (GRA) – PE; Bonito (BON) – PE; Toritama (TOR) – PE; Saloá (SAL) – PE; Buíque (BUI) – PE; Lagoa do Carro (LC) – PE; Surubim (SUR) – PE; Bezerros (BEZ) – PE; Serra Talhada (ST) – PE; Ouricuri (OUR) – PE; Juazeiro do Norte (JN) – CE; Picos (PIC) – PI; Sobradinho (SOB) – BA; Itaberaba (ITAB) – BA; and Rio de Contas (RC) – BA. Circles indicate populations with karyotyped individuals. Stars indicate populations with karyotyped individuals also analyzed for population genetics.

Specimens of *R. brasiliensis* (Bruner 1904) were collected from 21 populations in the Brazilian Northeast, of which 13 were located in the state of Pernambuco (PE), one in Sergipe (SE), one in Alagoas (AL), one in Paraíba (PB), one in Ceará (CE), one in Piauí (PI), and three in Bahia (BA) (Figure 1). Of these populations, 11 have been previously sampled and analyzed with regard to the occurrence and prevalence of B chromosomes by Loreto *et al.* (2008a) and Oliveira *et al.* (2011).

The grasshoppers were collected with the help of an entomological net and transported to the Laboratório de Biodiversidade e Genética de Insetos (LBGI), Instituto de Ciências Biológicas da Universidade de Pernambuco. The collections were permitted by ICMBio/SISBIO under the license number 16278-1.

### Conventional staining and C-banding

The karyotypes of 590 adult male specimens of *R. brasiliensis* were analyzed by conventional staining. First, the classic technique of testicular follicle squashing was performed, consisting in the maceration of the gonads in one drop of 45% acetic acid. Subsequently, the slides were subjected to heat of 65 °C for approximately 3 min for fixation of the chromosome preparation onto the slide, then stained with 5% Giemsa. The C-banding technique was performed according to Sumner (1972), with duration of exposure to the basic solution [Ba(OH)_2_] altered to 2 min, in individuals carrying B chromosomes from the populations of VIT and LC (Pernambuco – PE).

### Analysis of occurrence and frequency of B chromosomes and statistical test

The occurrence, frequency, and distribution of B chromosomes were analyzed in a total of 1.274 specimens. Of these, 394 were individuals from 10 populations being analyzed for the first time (GOI–PE, LC–PE, SAL–PE, BUI–PE, OUR–PE, ARA–SE, MAC–AL, JP–PE, JN–PE and PIC–PI) and 196 were individuals from four populations with samplings being expanded (ITA–PE, VIT–PE, BEZ–PE and ST–PE). The data for the remaining specimens were obtained from the literature (Loreto *et al.*, 2008b; Oliveira *et al.*, 2011), and included to provide a wider sampling for this species.

Using the software OpenEpi 3.01, a statistical significance test was carried out based on Fisher's exact test in order to verify whether the frequency of B chromosomes differs significantly among the populations. Furthermore, using the same software, analysis of confidence intervals (CI) was performed to estimate the maximum and minimum number that the frequency of B chromosomes may reach in an analysis with a population size of 1.000.000 specimens.

### DNA extraction and ISSR marker amplification

The genomic DNA of *R. brasiliensis* specimens was extracted from the leg muscle according to protocol described by Sambrook and Russell (2001). Subsequently, the DNA was purified through enzymatic digestion with RNase (10 mg/mL) for 1 h at 37 °C. Next, the concentration and quality of the extracted DNA were verified via electrophoresis in 1% agarose gel by comparison with DNA from phage Lambda DNA/*Hind* III marker (Fermentas Life Sciences).

Polymerase chain reaction (PCR) amplification was performed from a total volume of 10 μL containing 5 ng of DNA, 1X PCR buffer, 5 mM of MgCl_2_, 0,2 mM of dNTPs (2,5 mM), 1 pmol of primer (0,2 mM) and 1 U *Taq* polymerase (Invitrogen). The reactions were carried out in a thermocycler (Biosystems) as follows: Standard program at 94 °C for 4 min; 35 cycles at 94 °C for 30 s; annealing step (with temperature varying for each primer) for 45 s; extension at 72 °C for 2 min; and final extension at 72 °C for 7 min. The PCR products were visualized in 1,8% agarose gel using a transilluminator (Gel Logic/Carestream MI SE).

A total of 49 ISSR primers (Biotechnology Laboratory, University of British Columbia, Canada) were tested in one *R. brasiliensis* individual, with 33 presenting high numbers of amplified DNA fragments with good resolution. Selection of the most informative primers was accomplished by applying the 33 pre-selected primers to a sample of nine specimens from three populations (PIC–PI, JN–CE and JP–PB). Based on the genotyping, the data were grouped into a binary matrix of presence and absence. The analysis of the most informative primers was carried out according to the parameters number of total, monomorphic and polymorphic DNA fragments, and polymorphism indices: Polymorphism Information Content (PIC) (Roldán-Ruiz *et al.*, 2000), Marker Index (MI) (Prevost and Wilkinson, 1999) and Resolving Power (RP) (Varshney *et al.*, 2007). Furthermore, the Pearson correlation was calculated among the PIC, MI, and RP indices. The seven most-informative primers (807, 835, 844, 845, 846, 857, 866) were applied to 200 individuals from 10 populations, with 20 obtained from each population, thus representing the wide distribution of the species in the Northeast of Brazil (Figure 1, Table 1).

**Table 1:** State, mesoregion, altitude and geographic coordinate for the sampled populations of *Rhammatocerus brasiliensis* in the Northeast of Brazil.

State/ Mesoregion	Locality	Altitude	Geographic coordinates	Predominant biome / sampled area
Pernambuco/ Metropolitana do Recife	ITA - PE	3 m	07°45’0”S; 34°05’10”W	Atlantic forest - Mangrove/open area
	GOI - PE	13 m	07º33’38”S; 35º00’9”W	Atlantic forest - Restinga/open area
Pernambuco/ Zona da Mata	VIT - PE	162 m	08º07’35”S; 35º18’27”W	Atlantic forest/open area
	LC - PE	128 m	07º50’56”S; 35º19’14”W	Atlantic forest/open area
Pernambuco/ Agreste	GRA - PE	447 m	08º12’04”S; 35º33’53”W	Atlantic forest - Brejo de altitude/open area
	SAL - PE	745m	08º58’33”S; 35º41’15”W	Caatinga/open area
	BON - PE	443 m	08º28’13”S; 35º43’43”W	Atlantic forest - Brejo de Altitude/open area
	BUI - PE	798 m	08º37’23”S; 37º09’21”W	Atlantic forest - Brejo de Altitude/open area
	SUR - PE	394 m	07º59’31”S; 38º17’54”W	Caatinga/open area
	BEZ - PE	470 m	08º14’00”S; 35º47’49”W	Atlantic forest - Brejo de Altitude/open area
	TOR - PE	349 m	08°00’24”S; 36°03’24”W	Caatinga/open area
Pernambuco/ Sertão	ST - PE	444 m	07º49’59”S; 35º45’17”W	Caatinga/open area
	OUR - PE	451 m	07º52’57”S; 40º04’54”W	Caatinga/open area
Aracaju/ Leste Sergipano	ARA - SE	4 m	10º59’07”S; 37º04’24”W	Atlantic forest - Restinga/open area
Alagoas/ Leste Alagoano	MAC - AL	4 m	09º39’59”S; 35º44’06”W	Atlantic forest - Restinga/open area
Paraíba/ Mata Paraibana	JP - PB	37m	07º06’54”S; 34º51’47”W	Atlantic forest - Restinga/open area
Ceará/ Sul Cearense	JN - CE	377 m	07º12’47”S; 39º18’55”W	Caatinga/open area
Piauí/ Sudeste Piauiense	PIC - PI	206 m	07º04’37”S; 41º28’01”W	Caatinga/open area
Bahia/ Vale São Franciscano	SOB - BA	0 m	09º27’19”S; 40º49’24”W	Caatinga/open area
Bahia/ Centro-Sul	RC - BA	999 m	13º34’44”S; 41º48’41”W	Caatinga/open area
Bahia/ Centro-Norte	ITAB - BA	265 m	12º31’39”S; 40º18’25”W	Caatinga/open area

### Reproducibility test

The reproducibility of the ISSR markers was verified through analyses based on two independent PCR amplifications for all seven primers that were applied to all individuals of the studied populations. Non-reproducible *loci* were excluded from the genetic analysis.

### Genetic diversity and population structure

The genetic diversity index (GD), mean expected heterozygosity (H_E_), Wright's fixation index (F_*ST*_) and molecular variance (AMOVA) were calculated with the software Arlequin 3.5 (Excoffier and Lischer, 2010). The number of migrants (Nm) and coefficient of genetic differentiation (G_*ST*_) were calculated in the software Popgen 1.31 (Yeh *et al.*, 1999). The level of isolation by distance (IBD) was analyzed from the correlation between genetic and geographical distances (Mantel test, Sokal and Rohlf, 1995) using the software Arlequin 3.5 (Excoffier and Lischer, 2010), based on 10.000 permutations. At last, the population model most adequate to the data was established with the software Hickory 1.1 (Holsinger *et al.*, 2002) considering four possible models: i) full model, where the values of population differentiation (theta, θ, an analog of Wright's parameter F_*ST*_) and inbreeding (, an analog of F_*IS*_) differ from zero; ii) model =0, which assumes absence of inbreeding within the populations; iii) model theta=0, based on the absence of population differentiation; and iv) the free model, where the values are chosen randomly, independent of *a priori* information. The choice of the most suitable model was based on the parameters deviance information criterion (DIC), an analog of the Akaike information criterion (AIC) in the selection via Bayesian models, and Dbar, a measure of the level of adjustment with which the model adapts to the analyzed data (the lower the value of both, the better).

### Number of genetic clusters

The genetic clusters were analyzed with the software Structure 2.3.1 (Pritchard *et al.*, 2000) using the Bayesian algorithm, where the K-value was identified and analyzed to determine the level of genetic mixture among the populations. For each value of K (from 1 to 10), 10 independent rounds were performed with 600.000 replications and burn-in of 60.000 interactions. Finally, we used the website Structure Harvester (Earl, 2012), which implements the method of Evanno *et al.* (2005), to estimate the number of genetic groups (K) which best adjust the data.

## Results

### Cytogenetic data

**Figure 2:**
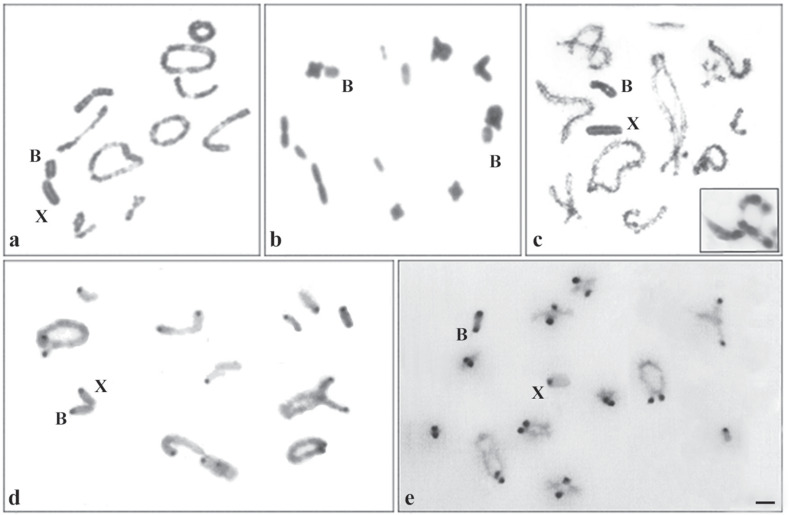
Meiotic cells of the species *Rhammatocerus brasiliensis* conventionally stained (a, b, c) and C-banded (d, e). a, c, d, e) Diplotene stage with one B chromosome. b) Diakinesis with two B chromosomes. c) Observe the variant B and the insect with three variant B chromosomes. Bar = 5 μm.

The karyotype *2n* = 23, X0, described for the species *R. brasiliensis*, was observed in 1.162 specimens. All chromosomes of the complement are acrocentric and were categorized, by size, into large (G1 – G3), medium (M4 – M8) and small pairs (P9 – P11). In addition, *2n* = 24, X0 + B was seen in 107 individuals, with the B chromosome being acrocentric and of medium size (Figures 2a,b,d) in all populations exhibiting this polymorphism (ITA–PE, GOI–PE, VIT–PE, LC–PE, GRA–PE, SAL–PE, BON–PE, BUI–PE, SUR–PE, BEZ–PE, TOR–PE, ST–PE, OUR–PE, MAC–AL, JN–CE, PIC–PI, SOB–BA, RC–BA, and ITAB–BA); *2n* = 25, X0 + 2Bs (Figure 2b) was observed in four specimens from ITA–PE, BEZ–PE, and OUR–PE; and *2n* = 26, X0 + 3Bs in one individual from ITA–PE (Figure 2c, insert) (Table 2)*.* Two distinct B chromosomes were observed: one verified in 19 of the 21 analyzed populations, of medium size and smaller than X. Another, here denoted as variant, was identified in the populations from ITA, BEZ and LC, with size similar to that of X and presence of a gap in the long arm (Figure 2c). In specimens with a single B chromosome, association with the X chromosome was observed (Figure 2a), whereas in the case of two B chromosomes, the association occurred with autosomes instead (Figure 2b).

**Table 2:** Occurrence, distribution, frequency and confidence interval of B chromosomes in the analyzed populations of *Rhammatocerus brasiliensis* in the Northeast of Brazil.

Locality	Individuals 1B	Individuals > 1B	Total	Prevalence (%)	Confidence Interval 95%	Number of individuals analyzed / reference
ITA - PE	8	2	73	13.69	7.2 - 23%	27/ Loreto *et al.*, 2008b; 46 / present study
GOI - PE	4		36	11.11	3.6 - 25%	Present study
VIT - PE	11		101	10.89	5.8 - 18%	57/ Oliveira *et al.*, 2011; 44 / present study
LC - PE	4		27	14.8	4.9 - 32%	Present study
GRA - PE	15		84	17.85	10 - 28%	Loreto *et al.*, 2008b
SAL - PE	1		43	2.32	0.12 - 11%	Present study
BON - PE	8		64	12.5	6 - 22%	Loreto *et al.*, 2008b
BUI - PE	1		27	3.7	0.18 – 16%	Present study
SUR - PE	11		147	7,48	4 - 13%	Oliveira *et al.*, 2011
BEZ - PE	15	2	142	11.97	7.4 - 18%	88/ Loreto *et al*., 2008b; 54 / present study
TOR - PE	6		66	9.1	3.8 - 18%	Loreto *et al.*, 2008b
ST - PE	9		144	6.25	3.1 - 11%	12/ Loreto *et al.*, 2008b; 80/ Oliveira *et al.*, 2011 and 52 / present study
OUR - PE	1	1	46	4.35	0.7 - 14%	Present study
ARA - SE	0		10	−	0 - 26%	Present study
MAC - AL	2		63	3.17	0.5 - 10%	Present study
JP - PB	0		35	−	0 - 8.2%	Present study
JN - CE	3		61	4.92	1.3 - 13%	Present study
PIC - PI	2		46	4.35	0.7 - 14%	Present study
SOB - BA	2		30	6.7	1.1 - 20%	Loreto *et al.*, 2008b
RC - BA	2		13	15.4	2.7 - 42%	Loreto *et al.*, 2008b
ITAB - BA	3		16	18.8	5 - 43%	Loreto *et al.*, 2008b
Total	107	5	1274			

The presence of B chromosomes was verified in almost all populations, with the exception of ARA–SE and JP–PB. Among populations with B chromosomes, ITAB–BA presented the highest frequency (18.8%) and SAL–PE the lowest (2.32%). The values for total frequency of males with B chromosomes in the populations varied from 0% to 18.8%, with a mean of 8.5% (Table 2). A significant difference existed in this regard between some populations (*p*-values > 0.05) identified by Fisher's pairwise exact test, namely: ITA–JP, 0.032; GRA–SUR, 0.031; GRA–ST, 0.012; GRA–OUR, 0.045; GRA–MAC, 0.008; GRA–JP, 0.007; GRA–JN, 0.031; GRA–PIC, 0.045; and BEZ–JP, 0.038 (Table 3).

**Table 3:** Statistical significance by Fisher's exact test between populations of *Rhammatocerus brasiliensis* analyzed for the presence of B chromosomes. Values of *p* < 0.05 are in boldface.

Locality	ITA	GOI	VIT	LC	GRA	SAL	BON	BUI	SUR	BEZ	TOR	ST	OUR	ARA	MAC	JP	JN	PIC	SOB	RC	ITAB
ITA-PE	−	0.961	0.739	0.633	0.625	0.077	0.999	0.287	0.220	0.871	0.562	0.119	0.173	0.510	0.057	0.032	0.152	0.173	0.517	0.999	0.851
GOI-PE		−	0.999	0.965	0.523	0.258	0.999	0.559	0.673	0.999	0.993	0.494	0.458	0.721	0.251	0.121	0.457	0.458	0.855	0.999	0.733
VIT-PE			−	0.908	0.252	0.154	0.936	0.462	0.480	0.962	0.921	0.285	0.326	0.671	0.128	0.065	0.306	0.326	0.778	0.908	0.586
LC-PE				−	0.315	0.658	0.762	0.350	0.999	0.765	0.999	0.999	0.947	0.999	0.694	0.371	0.977	0.947	0.999	0.783	0.519
GRA-PE					−	**0.017**	0.511	0.113	**0.031**	0.303	0.191	**0.012**	**0.045**	0.318	**0.008**	**0.007**	0.031	0.045	0.231	0.999	0.999
SAL-PE						−	0.121	0.999	0.396	0.093	0.314	0.569	0.999	0.999	0.999	0.999	0.900	0.999	0.732	0.262	0.113
BON-PE							−	0.375	0.360	0.999	0.731	0.217	0.255	0.587	0.100	0.051	0.236	0.255	0.642	0.999	0.764
BUI-PE								−	0.831	0.353	0.682	0.999	0.999	0.999	0.999	0.871	0.999	0.999	0.999	0.484	0.274
SUR-PE									−	0.275	0.876	0.855	0.722	0.945	0.387	0.177	0.739	0.722	0.999	0.570	0.287
BEZ-PE										−	0.720	0.138	0.217	0.587	0.067	**0.038**	0.188	0.217	0.633	0.985	0.656
TOR-PE											−	0.633	0.570	0.831	0.304	0.143	0.573	0.570	0.999	0.783	0.482
ST-PE												−	0.952	0.999	0.590	0.268	0.995	0.952	0.999	0.454	0.206
OUR-PE													−	0.999	0.999	0.638	0.999	0.999	0.999	0.415	0.206
ARA-SE														−	0.999	**0**	0.999	0.999	0.999	0.616	0.430
MAC-AL															−	0.821	0.969	0.999	0.775	0.266	0.107
JP-PB																−	0.503	0.638	0.418	0.138	0.053
JN-CE																	−	0.999	0.999	0.419	0.200
PIC-PI																		−	0.999	0.415	0.206
SOB-BA																			−	0.700	0.441
RC-BA																				−	0.999
ITAB-BA																					−

The C-banding procedure revealed positive C bands in the pericentromeric region of all chromosomes of the A complement, as well as in the B chromosomes (Figure 2d). An additional block was observed in the terminal region of the variant B chromosome (Figure 2e).

### Genetic diversity and population structure data

The seven ISSR primers used in the 10 analyzed populations generated a total of 95 reproducible fragments that varied in size between 300 and 2.500 base pairs (bp). Among the 95 analyzed loci, 71.05% were polymorphic, with the populations OUR–PE and ARA–SE presenting the highest and lowest percentages of polymorphism (90.5% and 13.6%), respectively (Table 4). In addition, the mean value for expected heterozygosity (H_E_) in the populations was 0.311, with OUR–PE and MAC–AL presenting the highest and lowest values, corresponding to 0.36 and 0.28, respectively (Table 4).

**Table 4:** Genetic diversity (GD) and mean heterozygosity (H_E_) estimated for the analyzed populations of *Rhammatocerus brasiliensis*.

Locality	H_E_	DG
ARA – SE	0.312	13.6
MAC – AL	0.285	68.4
JP – PB	0.320	83.1
ITA - PE	0.297	74.7
VIT – PE	0.329	50.5
BUI – PE	0.324	87.3
ST – PE	0.291	81.0
OUR - PE	0.363	90.5
JN – CE	0.290	82.1
PIC – PI	0.298	78.9
	0.311	71.0

The analysis of population models indicated the full model and =0 as the most adequate according to the parameters DIC and Dbar (Spiegelhalter *et al.*, 2002). However, the model =0, which assumes absence of inbreeding within the populations, is the most probable, owing to the gene flow evidenced by other analyses (F_*ST*_, AMOVA) and the dispersion capacity of *R. brasiliensis* (Table 5).

**Table 5:** Parameters estimated by the software Hickory for the model *f* = 0.

Parameter	Mean	S.D.	2.5%	97.5%
Theta-I	0.223	0.016	0.193	0.258
Theta-II	0.104	0.006	0.092	0.117
Theta-III	0.087	0.003	0.080	0.094
Theta-Y	0.132	0.018	0.101	0.172
Rho	0.591	0.041	0.512	0.673
Hs [ARA]	0.257	0.005	0.245	0.268
Hs [MAC]	0.257	0.005	0.246	0.268
Hs [JP]	0.270	0.005	0.259	0.281
Hs [ITA]	0.259	0.005	0.247	0.270
Hs [VIT]	0.293	0.005	0.282	0.304
Hs [BUI]	0.262	0.006	0.250	0.273
Hs [ST]	0.279	0.005	0.268	0.291
Hs [OUR]	0.290	0.005	0.279	0.301
Hs [JN]	0.279	0.005	0.269	0.290
Hs [PIC]	0.259	0.005	0.248	0.269
Hs	0.270	0.002	0.267	0.274
H_T_	0.298	0.002	0.294	0.302
G_ST_B	0.092	0.004	0.084	0.100

Molecular variance (AMOVA) reached 15.32% among the populations and 64.68% within them. Moreover, low genetic differentiation and high gene flow were observed upon analysis of the fixation index, F_*ST*_ =0.15; the coefficient of genetic differentiation, G_*ST*_ = 0.17; and the number of migrants, Nm=2.3869. Most of the pairwise F_*ST*_ values were low, though significant values were obtained for the populations JN–CE and MAC–AL, ST–PE and ITA–PE, ST–PE and BUI–PE, and JN–CE and ST–PE (Table 6). The Mantel test revealed lack of correlation between the genetic and geographical distances (r: 0.023121; P: 0.828000), which indicates the absence of IBD.

**Table 6:** Mantel test based on pairwise F_*ST*_ in 10 analyzed populations of *R. brasiliensis*.

Locality	ARA - SE	MAC - AL	JP - PB	ITA - PE	VIT - PE	BUI - PE	ST - PE	OUR - PE	PIC - PI	JN - CE
ARA - SE	−	208 km	488 km	437 km	374 km	260 km	360 km	475 km	488 km	652 km
MAC - AL	0.113	−	292 km	217 km	165 km	196 km	342 km	523 km	480 km	695 km
JP - PB	0.074	0.112	−	070 km	112 km	300 km	388 km	600 km	497 km	726 km
ITA - PE	0.184	0.183	0.129	−	057 km	269 km	381 km	589 km	499 km	729 km
VIT - PE	0.145	0.128	0.123	0.183	−	216 km	334 km	533 km	458 km	694 km
BUI - PE	0.166	0.161	0.077	0.113	0.203	−	142 km	326 km	284 km	500 km
ST - PE	0.156	0.147	0.171	0.240	0.124	0.226	−	197 km	138 km	363 km
OUR - PE	0.154	0.165	0.119	0.154	0.113	0.146	0.159	−	124 km	163 km
JN - CE	0.179	0.229	0.139	0.130	0.182	0.162	0.224	0.162	−	232 km
PIC -PI	0.128	0.178	0.098	0.127	0.134	0.164	0.191	0.116	0.098	−

**Figure 3:**
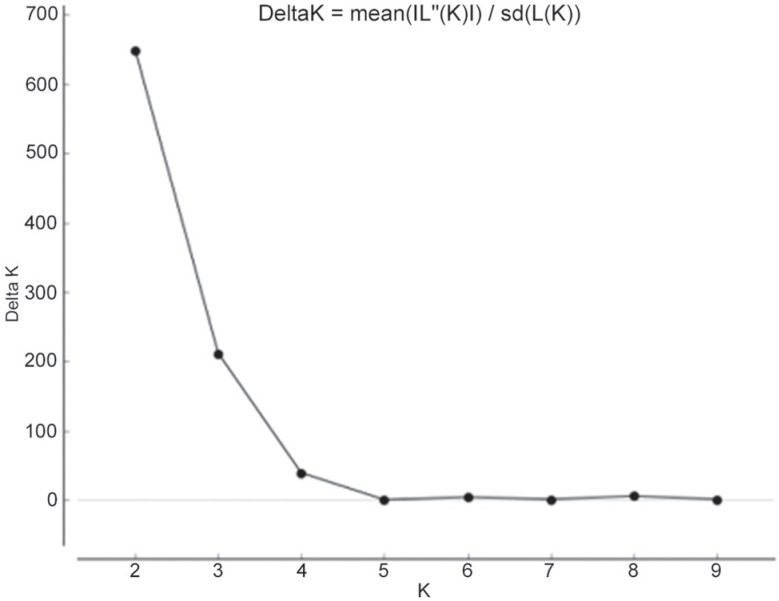
Delta K values according to the method of Evanno *et al.* (2005). Note the highest peak of K = 2.

**Figure 4:**
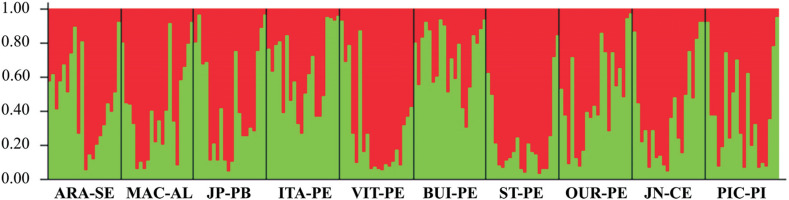
Ancestry of *Rhammatocerus brasiliensis* specimens traced by the software Structure version 2.3.1, using seven ISSR primers and 95 polymorphic bands. Each vertical bar corresponds to one of the 200 analyzed individuals and its population of origin. Bar length is proportional to parentage values inferred in each group for each individual.

The genetic clustering analysis performed with the software Structure presented K=2 as highest value, according to the method described by Evanno *et al.* (2005) (Figure 3), with two genetic groups being observed in all analyzed populations (Figure 4).

## Discussion

### B chromosomes in *R. brasiliensis*

The polymorphism of the B chromosome in *R. brasiliensis* is well represented in Acrididae grasshoppers (Palestis *et al.*, 2010). The broad distribution of *R. brasiliensis* individuals carrying B chromosomes suggests that this polymorphism is widespread among the populations, apparently there is a relation between the higher frequency of Bs and the geographical distribution, for example in GRA-PE, BEZ-PE, SUR-PE and VIT-PE. This tendency is likely associated with positive gene flow among those populations (better discussed below). Moreover, studies in other grasshopper species such as *Eyprepocnemis plorans* (Camacho *et al.*, 1980), *Trimerotropis pallidipennis* (Confalonieri, 1992) and *Dichroplus elongatus* (Remis, 1989) reported an increase in the frequency of recombination and chiasmata in host species, resulting in an advantageous increase in genetic variability (Camacho *et al.*, 1980, 2002; Riera *et al.*, 2004), which may be one of the reasons for the wide distribution of B chromosomes in *R. brasiliensis*.

The frequency of B chromosomes in *R. brasiliensis* was considered as high when above 11%, and as low when below 5%. However, all populations had values below 25%, which is indicative of the phase in which the B chromosome is in the life cycle (Camacho *et al.*, 1997; Araújo *et al.*, 2001). Given the values of the observed frequencies, the B of *R. brasiliensis* populations is probably in the stage of near neutrality of its life cycle. This is the longest phase, with successive generations being necessary to disperse the B chromosome, which has strictly vertical transmission. These frequencies may arise from stability, considering that these chromosomes tend to reach a balanced frequency over the years due to the efforts of the host genome to extinguish the B chromosome (Camacho *et al.*, 1997; Riera *et al.*, 2004).

The non-detection of B chromosomes in JP–PB and ARA–SE may be related either to elimination of the polymorphism or to low frequency in these populations as a manner of defense of the host genome, which attempts to get rid of parasite chromosomes (Cabrero *et al.*, 2017). Most of the analyzed populations did not show a significant difference with regard to the occurrence and frequency of B chromosomes. This may be associated to the low frequency of B chromosomes in several populations and/or a relatively low number of sampled populations lacking these chromosomes. The sampling number of these groups was possibly insufficient to detect the occurrence of B chromosomes, a fact that was corroborated by the confidence interval calculation, which indicated maximum frequency of 8.2% in JP–PB and 26% in ARA–SE. In *R. brasiliensis*, the different frequencies may be associated with the sum of several other factors, such as the number of generations since the origination or emergence of the B chromosome; population differences in the accumulation of the B chromosome, in case this polymorphism brings an advantage to the host in a population; and action of genetic drift (in particular for ARA–SE, based on its low genetic diversity; Table 4), which may be eliminating individuals carrying B chromosomes.

The occurrence of *R. brasiliensis* individuals with two B chromosomes in the populations of BEZ–PE, OUR–PE and ITA–PE, as well as three B chromosomes in ITA–PE, is also observed in some Acrididae, for instance *Aeropus sibiricus* (Linnaeus, 1767) with four acrocentric B chromosomes (Jetybayev *et al.*, 2018) and *Eumastusia koebelei koebelei* (Rehn, 1909), with two acrocentric B chromosomes (Anjos *et al.*, 2016). The presence of more than one B chromosome in *R. brasiliensis* is probably due to the non-disjunction of its sister chromatids during meiosis, as suggested by Camacho (2005). Some mechanisms may be acting in this phenomenon, such as a control region of non-disjunction located in the B chromosome itself (in the terminal or pericentromeric region), as already observed in rye (Endo *et al.*, 2008; Banaei-Moghaddam *et al.*, 2012). Moreover, non-disjunction of B chromatids could involve B-specific products (proteins or ncRNAs) that could associate with the centromeric regions and retard the separation during anaphase, resulting in both B chromatids ending up in the gamete (Benetta *et al.*, 2019). Various B-chromosome transcripts related to genes encoding proteins or pseudo-genes in rye (Banaei-Moghaddam *et al.*, 2013), fish (Valente *et al.*, 2014; Ramos *et al.*, 2016), fly (Bauerly *et al.*, 2014) and cervids (Makunin *et al.*, 2016) provide the basis to hypothesize about the involvement of protein-coding genes or pseudogenes in non-disjunction control. The constitutive heterochromatin (CH) may also act on the cohesion of the sister chromatids of the B chromosome, leading to non-disjunction (Banaei-Moghaddam *et al.*, 2012). However, the CH of B chromosomes from *R. brasiliensis* observed in this study, as well as by Loreto *et al.* (2008a) and Milani *et al.* (2018), is restricted to the pericentromeric and/or terminal region, indicating that other of the mechanisms listed above may be acting on the non-disjunction of the B chromosomes.

The small amount of CH restricted to the pericentromeric region of the B chromosomes from *R. brasiliensis* is different from that observed in other grasshopper species that possess B chromosomes with CH amplification rich in satellite DNA (Loreto *et al.*, 2008a; Bernardino *et al.*, 2017; Milani *et al.*, 2018). However, the hypothesis cannot be discarded that other repetitive DNA segments may be dispersed in euchromatic regions, such as transposable elements (TEs), which can be present in both observed types of B chromosomes (common and variant). TEs may have accumulated in the B chromosome and promoted the amplification of sequences (Marques *et al.*, 2018), contributing to the generation of the variant B chromosome.

B chromosomes can be differentiated with the C-banding technique based on the patterns of CH distribution, as observed in different types of B chromosomes in the grasshopper *E. plorans* (Henriques-Gil *et al.*, 1984). In addition, the terminal region of the variant B chromosome found in the population from LC–PE was also C-positive, which further contributes to the characterization of the variant B chromosome. This variant is probably undergoing the regeneration stage of the cycle, where it has accumulated modifications in order to escape neutralization by the host genome (Henriques-Gil and Arana, 1990; Camacho *et al.*, 1997). This way, the hypothesis of non-recent origin of B chromosomes is reinforced, since successive generations are necessary for the B chromosome to be nearly neutralized, accumulate modifications and scatter in the populations (Henriques-Gil and Arana, 1990; Camacho *et al.*, 1997).

### Genetic connectivity between populations of *R. brasiliensis*

As previously observed in other organisms (Taylor *et al.*, 2011; Pinheiro *et al.*, 2012; Izzatullayeva *et al.*, 2014), the ISSR marker was polymorphic in *R. brasiliensis*, with 71% of the loci presenting variation in the ten analyzed populations. The expected heterozygosity (H_E_) presented variation between 0.285 in MAC–AL and 0.363 OUR–PE. These values are similar to those described for other insects, such as the grasshoppers *Pezotettix giornae* (Rossi, 1794) (Gauffre *et al.*, 2015) and *E. plorans* (Manrique-Poyato *et al.*, 2013a); the honey bee *Apis mellifera meda* (Skorikow, 1829) (Rahimi *et al.*, 2016); as well as the low value in the beetle *Canthon staigi* (Pereira, 1953) (Ferreira-Neto *et al.*, 2017) and the high value in *Lucanus cervus* (Linnaeus, 1758) (Snegin, 2014). Although it has already been evinced that in grasshoppers the presence of B chromosomes may contribute to the increase in genetic variability, this relationship was not observed for *R. brasiliensis*, in which some populations present low H_E_, such as ST–PE (0,291) and JN–CE (0.290). Moreover, the total expected heterozygosity (H_T_= 0.298) was greater than the subpopulation heterozygosity (H_S_= 0.270), indicating that there may be a deficit of heterozygotes.

The data generated in this study suggest that the occurrence of B chromosomes in *R. brasiliensis* is related to its population genetic structure, given the wide dispersion of the B chromosome verified in the karyotyped individuals, positive gene flow (Nm = 2.3869), and low genetic differentiation F_*ST*_ (0.15) and G_*ST*_ (0.17) among the analyzed populations. Owing to the migratory behavior of *R. brasiliensis* (Carbonell, 1988), this is an expected pattern, considering that the probable means of dispersion of the B chromosome in different geographic ranges, according to Camacho *et al.* (2015), would be through gene flow between hosts and individuals from populations that do not have the B chromosome. The Mantel test suggested a lack of correlation between genetic and geographical distance, even between populations more than 700 km apart (ITA–PE and JN–CE). This may be contributing to the dispersion of the B chromosomes, as their transmission is strictly vertical (Munoz *et al.*, 1998). Pairwise F_*ST*_ indicated an overall low genetic differentiation; however, some populations presented F_*ST*_ with significantly high values (JN–CE and MAC–AL, JN–CE and ST–PE, ST–PE and ITA–PE, ST–PE and BUI–PE; Table 6).

The data from molecular variance (AMOVA), which was greater within the populations (84.68%), when associated to Structure data, corroborate the low indices of genetic differentiation, where the presence of a mixture of two genetic profiles is related to migratory habit, wide mobility, and flight capacity of *R. brasiliensis* (Carbonell, 1988, 1995). Native grasses, for instance *Trachypogon sp.* (Poaceae), constitute the preferred food of grasshoppers (Silva *et al.*, 2006). Therefore, the large offer of grasses in the localities sampled in this study and the absence of effective geographic barriers between the populations are fundamental factors for the wide dispersion of the analyzed specimens. The feeding preference of *R. brasiliensis* is, which is strongly related to its mandibular structure of graminivorous type, is also present in other Gomphocerinae such as *Achurum carinatum* (Walker, 1870), *Dichromorpha viridis* (Scudder, 1862) and *Orphulella pelidna* (Burmaister, 1838) (Smith and Capinera, 2005). For other grasshopper species, for instance *Mioscirtus wagneri* (Eversmann, 1859) and *Ramburiella hispanica* (Rambur, 1838), a positive relationship was also observed between the distribution of plants used as food and the genetic connectivity between populations (Ortego *et al.*, 2010, 2015).

Although the populations of Pernambuco and other states occupy different altitudes (range 0 to 999 m), as well as potential geographic barriers (Borborema Plateau, Chapada do Araripe and São Francisco river), *R. brasiliensis* individuals seem to be capable to overcome these obstacles and maintain a positive gene flow among the analyzed populations. This way, no correlation was observed between the occurrence of B chromosomes and the distribution of *R. brasiliensis* in different landscapes. For this species, the altitude of the analyzed populations did not seem to influence the frequency of B chromosomes either. This observation differs from that of Manrique-Poyato *et al.* (2015), who stated that individuals with B chromosomes have low tolerance to high-altitude environments as a result of delay in meiosis due to the presence of B chromosomes that may negatively select the host (Hewitt and East, 1978; Harvey and Hewitt, 1979).

Based on literature evidence that B chromosomes are only transmitted vertically, it is suggested that, for the species *R. brasiliensis*, the broad distribution of B chromosomes indeed arises from the extant gene flow among the analyzed populations. The migratory habit coupled to the high flying capacity of *R. brasiliensis*, besides the wide offer of food resources in the sampled localities, are factors that certainly contributed to the genetic connectivity of its populations, and consequently for the broad dispersion of the B chromosome. A variant B chromosome, with CH blocks in the pericentromeric and terminal regions, was detected and is probably currently in the regeneration stage of the cycle. The presence of one such variant B chromosome reinforces the hypothesis of the non-recent origin of the B chromosome, considering that successive generations are necessary for such a chromosome to be nearly neutralized, accumulate modifications and be dispersed among distinct populations.
